# Genome-Wide Survey for Biologically Functional Pseudogenes

**DOI:** 10.1371/journal.pcbi.0020046

**Published:** 2006-05-05

**Authors:** Örjan Svensson, Lars Arvestad, Jens Lagergren

**Affiliations:** Stockholm Bioinformatics Centre, Royal Institute of Technology, Albanova University Center, Stockholm, Sweden; Weizmann Institute of Science, Israel

## Abstract

According to current estimates there exist about 20,000 pseudogenes in a mammalian genome. The vast majority of these are disabled and nonfunctional copies of protein-coding genes which, therefore, evolve neutrally. Recent findings that a Makorin1 pseudogene, residing on mouse Chromosome 5, is, indeed, in vivo vital and also evolutionarily preserved, encouraged us to conduct a genome-wide survey for other functional pseudogenes in human, mouse, and chimpanzee. We identify to our knowledge the first examples of conserved pseudogenes common to human and mouse, originating from one duplication predating the human–mouse species split and having evolved as pseudogenes since the species split. Functionality is one possible way to explain the apparently contradictory properties of such pseudogene pairs, i.e., high conservation and ancient origin. The hypothesis of functionality is tested by comparing expression evidence and synteny of the candidates with proper test sets. The tests suggest potential biological function. Our candidate set includes a small set of long-lived pseudogenes whose unknown potential function is retained since before the human–mouse species split, and also a larger group of primate-specific ones found from human–chimpanzee searches. Two processed sequences are notable, their conservation since the human–mouse split being as high as most protein-coding genes; one is derived from the protein Ataxin 7-like 3 (ATX7NL3), and one from the Spinocerebellar ataxia type 1 protein (ATX1). Our approach is comparative and can be applied to any pair of species. It is implemented by a semi-automated pipeline based on cross-species BLAST comparisons and maximum-likelihood phylogeny estimations. To separate pseudogenes from protein-coding genes, we use standard methods, utilizing in-frame disablements, as well as a probabilistic filter based on Ka/Ks ratios.

## Synopsis

Svensson, Arvestad, and Lagergren conducted a genome-wide survey for and analysis of human pseudogenes, i.e., gene copies with lost protein-coding ability, with the aim of discovering biologically functional ones. Their main motivation was a 2002 *Nature* paper revealing in vivo functionality for a mouse Makorin pseudogene, Makorin1-p1. Their work is in line with extensive research in recent years concerning ncRNA. The method consists of a BLAST-based pipeline augmented by modern maximum-likelihood phylogeny estimations. Several examples of unknown genes and present in silico tests favoring the hypothesis that these are functional pseudogenes were found. In the result set, there are two examples from the Ataxin family; a poorly characterized gene family which, however, includes a number of genes related to neurogenerative disorders. A discovery of new members in this gene family should be of great interest to experimentalists in the field. To the best of our knowledge, functional pseudogenes have never been observed in humans. The results suggest, however, that while functional pseudogenes are relatively rare on a long evolutionary timescale, they nevertheless exist. These deserve attention, of course, similar to any other previously uncharacterised gene.

## Introduction

Pseudogenes are sequences of genomic DNA lacking the protein-coding capability of their paralogous counterpart [1,2]. A pseudogene can be arbitrarily similar to the original gene, but differ by the fact that it accumulates *disablements* (in-frame stop codons and sequence frameshifts), which protein-coding genes do not. Because pseudogenes are not protein-coding, they are often thought of as being without function and therefore released from selective pressure. The origin of a pseudogene is generally either a segmental duplication, or a retrotransposition where mature mRNA is reversely transcribed into cDNA and reinserted in a new genomic position. The resulting pseudogene is in the latter case called *processed* as compared with duplicated or *nonprocessed*.

Studies of pseudogene populations are often motivated by the dilemma that their similarity to ordinary genes constitutes for gene finders and hybridization experiments. Pseudogene sequences can, given their nonfunctionality, be viewed as a molecular fossil and have been used to measure background genomic substitution rates [3,4].

However, evidence has occasionally been found, in *Drosophila* and recently also in mouse [5], of pseudogene functionality, as well as of conservation (see [6] for a review). In [5], evidence is given for a regulatory role of the mouse Makorin1 pseudogene *Makorin1-p1*. It was proposed in [5] that the function of the transcribed pseudogene is to stabilize the Makorin1 mRNA. A follow-up study [7] established that Makorin1-p1 is in fact conserved across several mouse species, although it is not found in more distantly related species such as rat or human.

Several surveys [8–10] have located and annotated pseudogenes in the human and mouse genomes. Despite using slightly different pseudogene definitions and methodologies for finding them, they end up with similar numbers of human pseudogenes (altogether about 20,000 sequences out of which some 8,000 show evidence of processing). The authors of [11] used more restrictive criteria, and identified about 3,600 human processed pseudogenes. The main theme for these studies is that sequences sufficiently similar to a known protein sequence are considered potential pseudogenes. The final classification as pseudogene is based on proof of sequence disablements (primarily in-frame stop codons and sequence frameshifts), Ka/Ks values indicating neutral evolution, and, importantly, that the sequences are not overlapping any known gene.

In a recent article [12], the authors went further and looked specifically for human-transcribed processed pseudogenes. They found that some 4%–6% of all human processed pseudogenes could be confidently mapped to sequences in expression databases. The same group then continued with a more careful annotation of pseudogenes on Chromosome 22, utilising tiling microarray technology [13], concluding that this rate was probably an underestimate and that maybe as much as 1/5 of all pseudogenes are transcribed. Another investigation in the same spirit [14] found that the percentage of expressed pseudogenes differ significantly between human and mouse. They reported 2%–3% and 0.5%–1%, respectively, using the most restrictive criteria.

A human–mouse comparative study [12] concludes that the vast majority of transcribed human pseudogenes are lineage specific. Only some 5% were found to have potential orthologs in mouse.

That a pseudogene is transcribed is not sufficient evidence of biological function. To obtain functional candidates, we decided to look for conserved pseudogenes common to human and mouse, originating from one duplication predating the human–mouse species split and having evolved as pseudogenes since the species split. In cases where the species split occurred sufficiently early, strong conservation and ancient origin gives evidence of the potential functionality of the pseudogenes. We have developed a pairwise comparative genomics methodology based on an explicit evolutionary model, which focuses on pseudogenes common to the two lineages. We also test the potential functionality of the found pseudogenes using enrichment of transcription and synteny.

We describe our methodology using the example of a human–mouse comparison. Our procedure takes as input a quartet of sequences representing, respectively, a human gene, a corresponding human pseudogene, the orthologous mouse gene, and a corresponding mouse pseudogene, and analyzes how they have evolved. All four basic evolutionary scenarios that can occur with respect to duplication and gene-to-pseudogene transitions are described below. When analyzing how well a scenario describes the evolution of a quartet, different models of sequence evolution are used for gene and pseudogene lineages.

**Figure 1:**
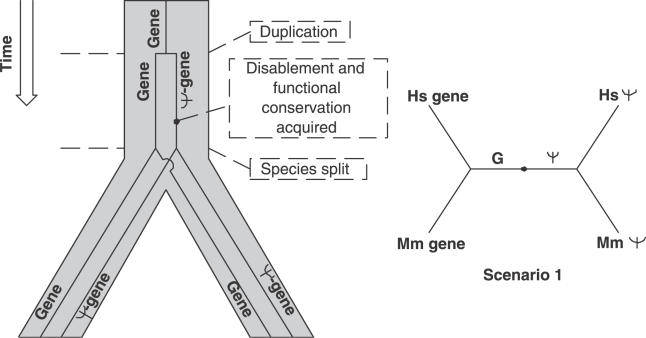
Evolutionary Scenario *S*1, Describing the Case where the Pseudogene Originated before the Species Split and Has Acquired as well as Maintained Function G and *ψ* on tree branches refer to gene and pseudogene evolution, respectively.

The first scenario, *S*1, is what we expect for conserved pseudogenes originating before the species split (see Figure 1). 

**Figure 2:**
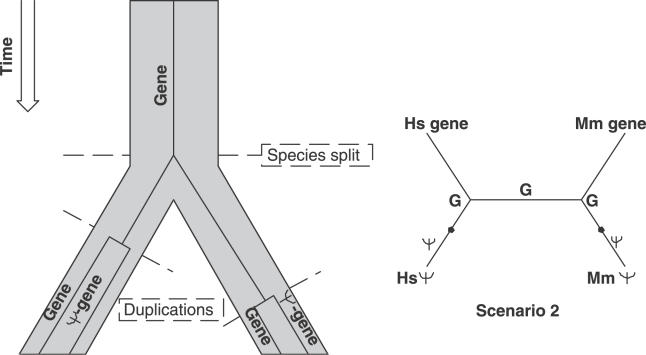
Evolutionary Scenario *S*2, Describing the Common Case of Late and Independent Pseudogene Origin

An alternative scenario, *S*2, is expected if both pseudogenes originated independently of each other, after the species split (see Figure 2). In our human–mouse comparison, the evolution of most quartets are best described by *S*2. A likely explanation for this is that dead-on-arrival pseudogenes [15] originating before the human–mouse species split have most often diverged beyond the limit of recognition. With approximately 0.5 substitutions per site, fewer than 10% of neutrally evolving genomic elements can be found using BLAST [16]. 

**Figure 3:**
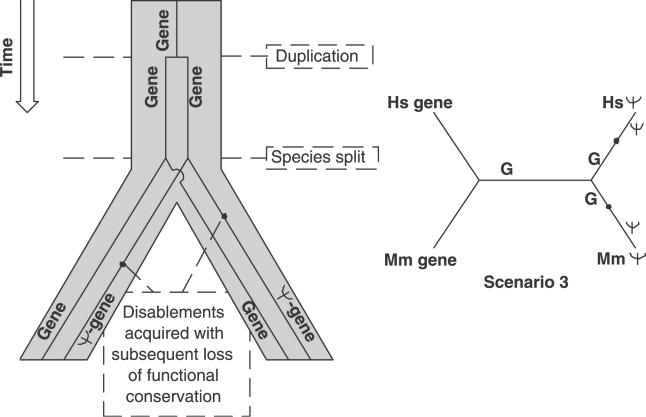
Evolutionary Scenario *S*3, Describing Independent Transitions

The third scenario, *S*3 (see Figure 3), is similar to *S*1. The difference is that the transition from gene to pseudogene occurred subsequent to the species split. This could mean that a pair of pseudogenes was in fact functional genes prior to the transition, but has since then evolved without selective pressure.

A fourth scenario where the human gene has the mouse pseudogene as a sibling in the gene tree is conceivable. We have never observed this scenario. 

We have applied our comparative methodology to human–mouse as well as to human–chimpanzee and found the first examples of human pseudogenes showing signs of functionality.

## Results

We started with the 12,687 presumably orthologous protein pairs retrieved (see Materials and Methods) from the Inparanoid web site [17] for which gene sequences could be found. We used BLAST to scan the human and mouse genomes for potential pseudogenic sequence pairs (see Materials and Methods). A pseudogene pair corresponding to a protein pair was then used together with the gene sequences to form the sequence quartets on which we base our analysis.

This initial search with subsequent refinement resulted in 168,855 such quartets. For the vast majority of these quartets, one or both pseudogene sequences overlap regions of *known* or *predicted* protein-coding genes. Gene position data from Ensembl were used to filter out known genes. Predicted genes are kept for further analysis, since it is known [8] that gene predictors sometimes mistake pseudogenes for protein-coding genes.

**Figure 4:**
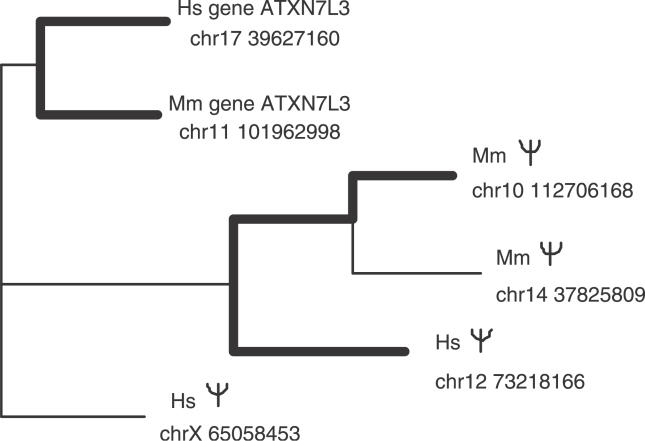
Visualization of the Effect of Mutual-Best-Hit Filtering The tree shows the evolutionary history for a sequence set associated with the ATXN7L3 orthologous proteins. We have here found two potentially pseudogenic sequences in each species and this gives us a total of four quartets to investigate; the gene-sequence pair together with any human–mouse combination of the pseudogenes. It is unlikely that the human chrX pseudogene is closely related to any of the mouse ones and therefore any quartet including the sequence from the X chromosome should be of limited interest. If we pair a particular human pseudogene only with the most similar mouse pseudogene (and vice versa), the sole remaining example is the human chr12–mouse chr10 pair.

The set that remains after filtering constitutes 11,146 sequence quartets originating from 1,349 protein pairs. The distribution of quartets per protein pair is highly nonuniform. While many gene pairs lack corresponding pseudogene pairs, a handful (EF12, G3PT, LDHB, TSY1, UB46, and several ribosomal genes) are origins of large pseudogene families in both species. Using the mutual-best-hit filtering outlined in Figure 4, we, however, removed a large number of pairs likely to be insignificant; after this step 1,453 sequence quartets remained. We divide these into four classes according to the following: class 1—pairs that have detectable disablements and do not overlap any Ensembl gene prediction; class 2—pairs that have detectable disablements and overlap an Ensembl gene prediction; class 3—pairs that do not have detectable pseudogenic disablements and do not overlap any Ensembl gene prediction; and class 4—pairs that do not have detectable pseudogenic disablements and overlap an Ensembl gene prediction.

We used the partition of our data induced by this classification in combination with mutual-best-hit filtering. The number of sequence quartets belonging to the classes are: class 1—247 quartets; class 2—299; class 3—146; and class 4—761 (see Table 1).

Our aim is to find those quartets for which *S*1 is the most likely explanation. We use a probabilistic methodology to compare scenarios (see Materials and Methods), to obtain *p*-values for any possible alternative hypothesis with respect to the interesting one, namely, that *S*1 best describes a given quartet. For visualization purposes, we also consider quotients of type *L*_1_/*L*_2_, where *L_i_* is the log-likelihood corresponding to scenario *i;* for a particular quartet, a value *L*_1_/*L*_2_ < 1 suggests that L_1_ is preferable to *L*_2_.

**Figure 5:**
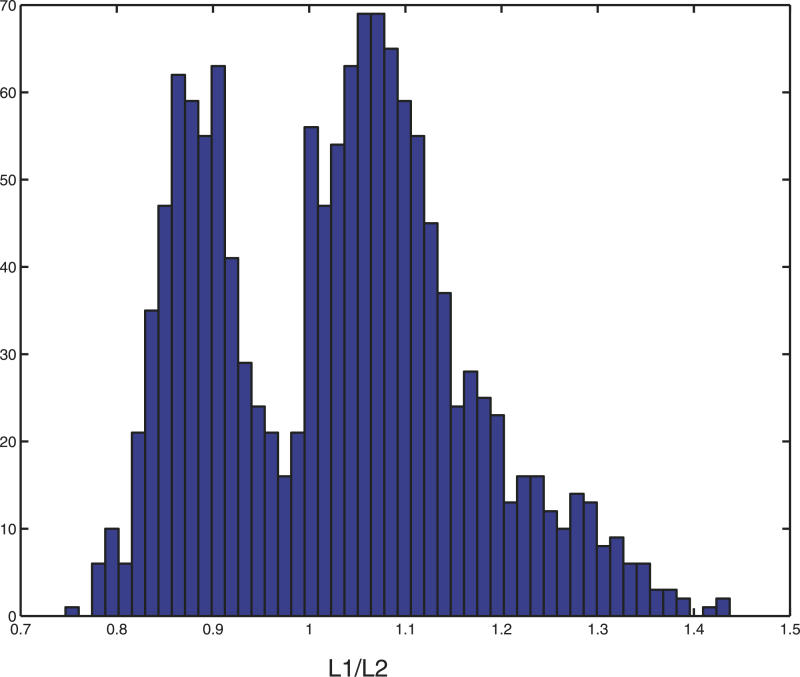
Histogram of Likelihood Quotients when Comparing Scenarios *S*1 and *S*2

For the majority of our 1,453 quartets, data support *S*2 (Figure 5), the scenario where pseudogenes originated later than the species split. In 425 out of 1,453 cases (29%), the *p*-value for *S*1 being the scenario that best explains our data is less than 0.001.

Interestingly, we note a bimodal pattern with one large hump distributed around 1.1 and another one distributed around 0.9. That is, in a large majority of cases, data show clear preference for *either S*1 *or S*2; it is only for a comparatively small number of cases that the quotients are close to 1.

**Figure 6:**
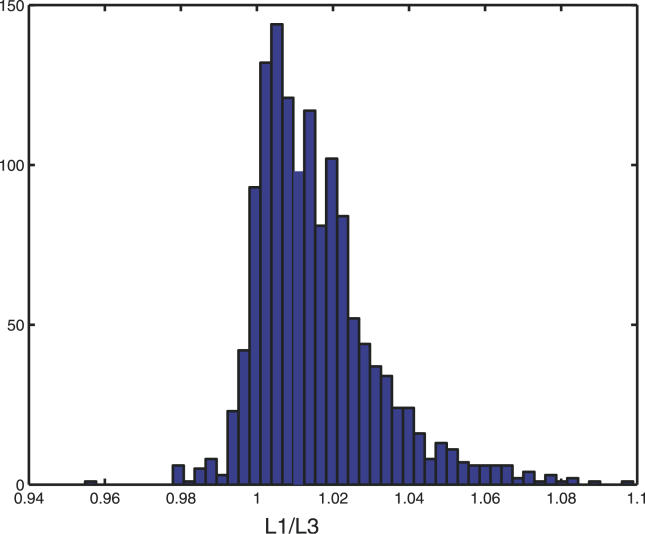
Histogram of Likelihood Quotients when Comparing Scenarios *S*1 and *S*3

We now use the same technique to compare *S*1 and *S*3. Remember that *S*3 is the scenario where the transitions from genes to pseudogenes were independent of each other and occurred *subsequently* to the speciation. Hence it is only the models of sequence evolution used for genes and pseudogenes, respectively, that distinguish *S*1 from *S*3. The likelihood values are in this case much less varied, yielding many quotients close to one (Figure 6).

For 73 of the 425 quartets, *S*1 is the explanation favored by our method and for 30 of these 73 the *p*-value is lower than 0.1. For 352 sequence pairs, *S*3 is the most likely topology, and 262 of those clearly favor *S*3 (*p*-value again lower than 0.1).

To summarize, we have 30 quartets for which the sequences suggest that: 1) the pseudogenes are evolutionarily conserved since before the human and mouse speciation; 2) they have been pseudogenes since prior to the speciation.

Because we find 30 such quartets, and the number of quartets expected to pass our scenario test is 1453 * 0.001 * 0.1 ≈ 0.15, it is reasonable to conclude that a significant number of these 30 quartets are ancient pseudogenes, i.e., satisfying 1) and 2). 

We are now going to investigate these 30 sequence quartets further, with the aim of testing their potential biological function. The criteria that will be our focus are synteny, expression evidence, and conservation.

### Synteny

Synteny can be used as a means to evaluate our methodology's capacity to separate S1 and S3 from S2 quartets. It is also interesting to compare the fraction of syntenic quartets among S1, S3, and genes. The latter can be seen as a test of functionality.

It has long been known that eukaryotic genomes undergo rearrangements on both microscopic (intrachromosomal with a span < 1 Mb) and macroscopic (intrachromosomal with larger span, as well as interchromosomal) level during evolution [18]. By using so-called sequence markers, often protein-coding segments, it has been possible to infer maps of syntenic regions, that is, regions of conserved marker order.

The orthologous pairs of protein-coding genes in our data set have the following synteny relations: 69% syntenic, 2% reversed syntenic, 11% corresponding chromosomes, 4% nonsyntenic, and 13% unknown synteny (see Materials and Methods). We find 20 out of the 30 *S*1 pairs in synteny and five are found close to synteny (Materials and Methods).

It is reasonable that sequences that have originated from duplication events prior to the species split (sequences belonging to *S*1 and *S*3 quartets) are primarily found in syntenic positions, as we have seen is the case for genes. Conversely, there is no reason to presuppose this for quartets showing preference for *S*2 (remember that the pseudogenes here are expected to have originated independently of each other). From inspection of Table 2, we see the following: out of our 30 *S*1 sequence pairs, 20 are syntenic (67%). A similar amount, 149 out of 262 (57%), of *S*3 sequences are found in syntenic regions. Only 130 out of the total 702 (19%) *S*2 sequence pairs are syntenic. In fact, one could argue that the latter percentage is unexpectedly high. A possible explanation is that these are a result of tandem duplications; that is, the duplicated sequences are found nearby the original ones and are therefore in synteny. The tendency for class 4 to be found in syntenic regions could simply be due to the fact that these are detected by comparative gene finders, which often use synteny as a criterion [19].

**Table 1:**
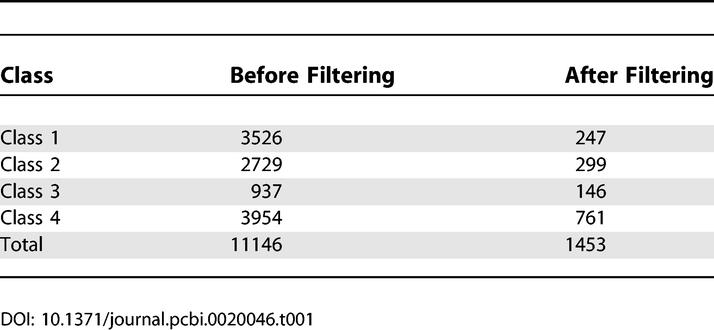
Number of Human–Mouse Sequence Pairs prior to and following Mutual-Best-Hit Filtering

**Table 2:**
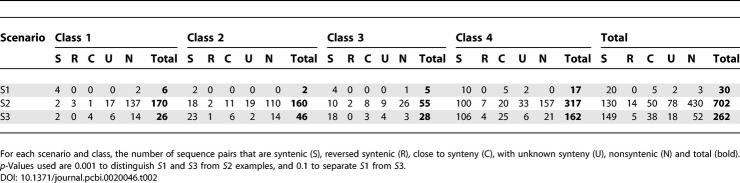
Number of Sequence Pairs in Each Class Favoring a Particular Scenario

It is notable that within classes 1, 2, and 3, ten of the 13 *S*1 pseudogene pairs are syntenic, while only 43 out of 100 *S*3 pairs are syntenic.

If we again consider the ATXN7L3 tree (Figure 4), we see that among the pseudogenes only the human Chr12/mouse Chr10 pair, the pair retained after mutual-best-hit filtering, is found in syntenic positions.

### Pseudogene Expression

We investigated whether our candidates for potential function are enriched for transcription or not, by searching publicly available databases for transcript sequences, expressed sequence tags (ESTs) and mRNAs. An EST or mRNA sequence is postulated to come from a specific pseudogene if its sequence is more similar to the pseudogene than it is to any other known gene or pseudogene (see Materials and Methods for details). We found that, out of the 30 sequence pairs showing preference for *S*1, 22 are transcribed in both human and mouse. For the 20 syntenic *S*1 sequence pairs, 17 are transcribed in both species and all but one are transcribed in either human or mouse (see Table 3). Notable are the ATX1 and ATXN7L3 duplicates, both class 1 members, for which we find ESTs from many different tissues (human thyroid, colon, and prostate among them), and also class 3 ZNF629 duplicates, each perfectly matched by approximately 1,000-bp-long mRNAs.

**Table 3:**
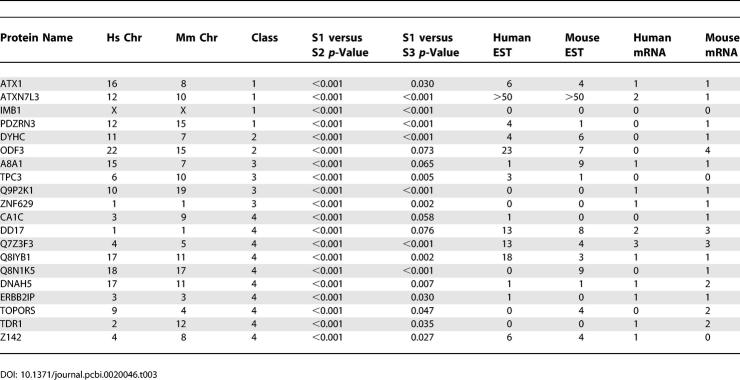
*p*-Values for Scenario Comparisons and Pseudogene Expression Evidence (Number of Matching EST and mRNA Sequences) for the 20 Syntenic *S*1 Quartets

Among the 20 syntenic S1 sequence pairs, the only completely unexpressed example is the IMB1 copy found on the X chromosome. This pair shows clear preference for *S*1, and is also unusually well-preserved for a nonfunctional pseudogene. This might indicate that the IMB1 pseudogene was functional for a short period after the species split, but it could also simply be an effect of the X-chromosome's lower mutation rate [20].

The majority of these pseudogenes are much less expressed than their respective genes (to the latter, one can generally map large numbers of ESTs originating from tissues throughout the body).

To perform an enrichment test we need a good comparison set. We believe that *S*3 contains many young pseudogenes, that is, those which recently underwent the transition from gene to pseudogene, but also protein-coding genes. It is reasonable to assume that young pseudogenes are more frequently transcribed than older ones. For these reasons, *S*3 is not a good comparison set, and no other such set is available either.

**Figure 7:**
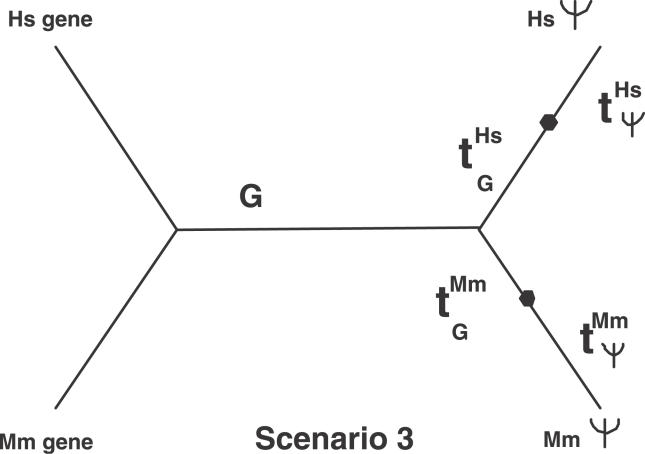
*S*3 Topology with Gene-to-Pseudogene Breakpoints 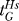
 refers to the length of the branch on which the human pseudogene has evolved genelike, and similarly for 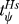 *,* 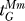 *,* and 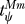 .

Instead, we focus on the correlation between the *S*3 pairs' positioning of the gene-to-pseudogene transitions (see Figure 7) and their pseudogene expression. For this we adopted the following labeling scheme with notation from Figure 7:


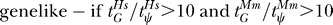



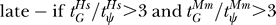



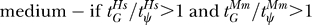



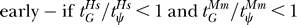



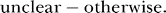


If we assume that the rate of pseudogene creation does not vary over time, then the low number of detected early pairs—only nine out of 198 non-unclear *S*3 examples conform to this group—is a sign that most pairs of the same age as the early pairs have diverged beyond recognition.

Table 4 shows the number of examples in each group together with an evaluation of their tendency to be expressed. We note that whereas in the genelike and late groups a large majority (94% and 80%, respectively) is expressed in both organisms, the figures are much lower for the early (22%) and unclear (44%) ones. We could also compare these figures with the corresponding figures for *S*1 examples where 22 out of 30 examples (73%) are expressed in both organisms. As can be seen in Table 4, this tendency is even more pronounced if we count only syntenic examples. It is reasonable to conclude that the majority of the early pairs are nonfunctional, because they are expressed to such a low extent. Considering the higher age and the extent of expression for the 20 *S*1 pseudogenes, it is also reasonable to conclude that this set contains pseudogenes of potential biological function.

**Table 4:**
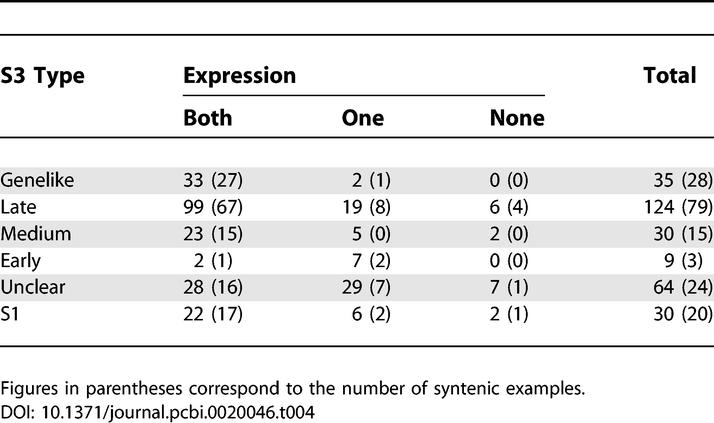
Human and Mouse Expression for 262 S3 Quartets Selected as Described in Table 1

### Conservation

According to estimates in [21], the neutral rate of substitution has been roughly 0.5 substitutions per site since the divergence of the human and mouse lineages. This estimate conforms to 67% sequence identity for orthologous regions under no selective pressure. At the other extreme, protein-coding sequences have, on average, approximately 85% conservation [21].

**Figure 8:**
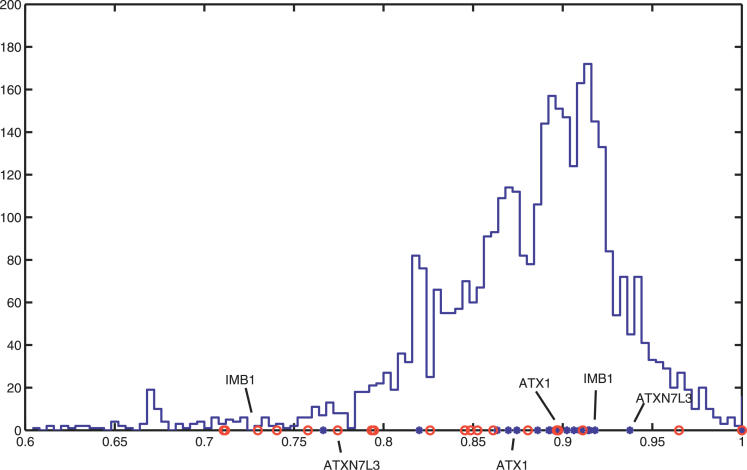
Conservation between Human and Mouse Gene and Pseudogene Sequences for the 20 Syntenic *S*1 Sequences Blue stars indicate genes. Red circles indicate pseudogenes. The histogram shows, for reference, the conservation of all genes giving rise to pseudogenes. Compare with Table 5, which lists the same data.

**Figure 9:**
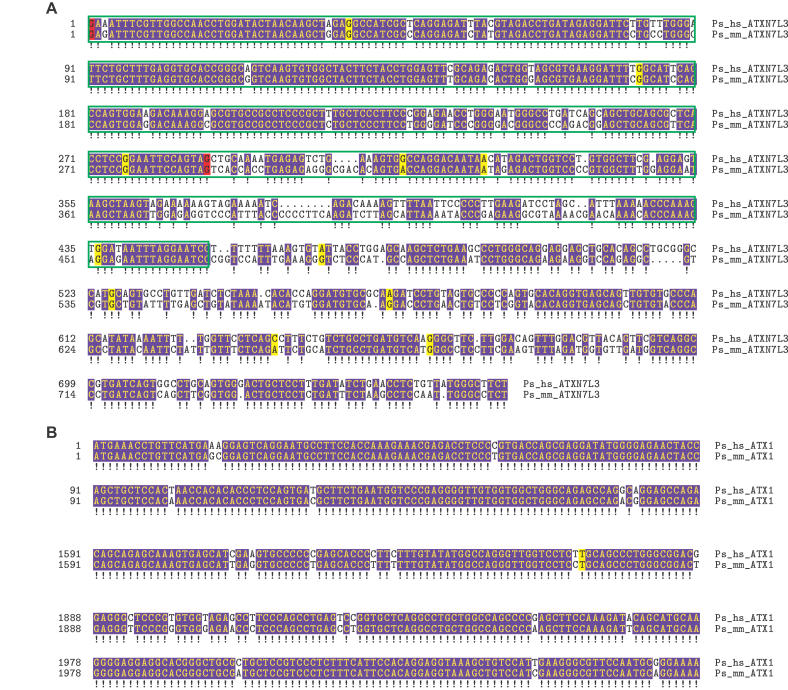
Recognizing Pseudogenes by Inspecting Their Alignment (A) An alignment, visualized with TeXshade [34], of the processed copies to the ATXN7L3 human and mouse protein-coding genes. The human as well as the mouse ATXN7L3 contains 12 exons, which are all present in the respective duplicates. Approximate exon borders are shown in yellow. The most interesting part consists of columns 1–468 (boxed green), which according to several EST and mRNA sequences is the only segment expressed. It consists of a highly conserved part, 1–288 (red), which is a potential open reading frame, followed by part 289–468 with pseudogenic disablements. (B) Selected parts of the alignment of the ATX1 copies which are also processed. The protein-coding genes contain eight exons of which only parts of the last two code for protein. The entire segment of the pseudogenes corresponding to the protein-coding parts of the genes is expressed. The possibility that the processed copies are protein-coding cannot not be completely ruled out, however. Indeed, each pseudogene consists of one single 2,068-bp-long open reading frame. However, the frame induced by the alignments to the protein-coding genes contains several pseudogenic disablements.

We will now address where our putatively functional pseudogenes are placed along that scale. We note (Figure 8 and Table 5) that although the conservation for the 20 syntenic *S*1 pseudogene pairs is not as strong as for the corresponding genes, it is in most cases significantly above the 67% limit (*p*-values are computed using Hoeffding's bound, see Materials and Methods). For instance, the ATX1 derivative shows conservation at least as high as a typical gene, even slightly higher than its paralogous gene (Figure 8). The ATXN7L3 duplicate, previously discussed, has conservation similar to that of a protein-coding gene. It is 77% conserved, counted over the total alignment, but a 288-bp-long section in the beginning is 89% conserved (Figure 9).

**Table 5:**
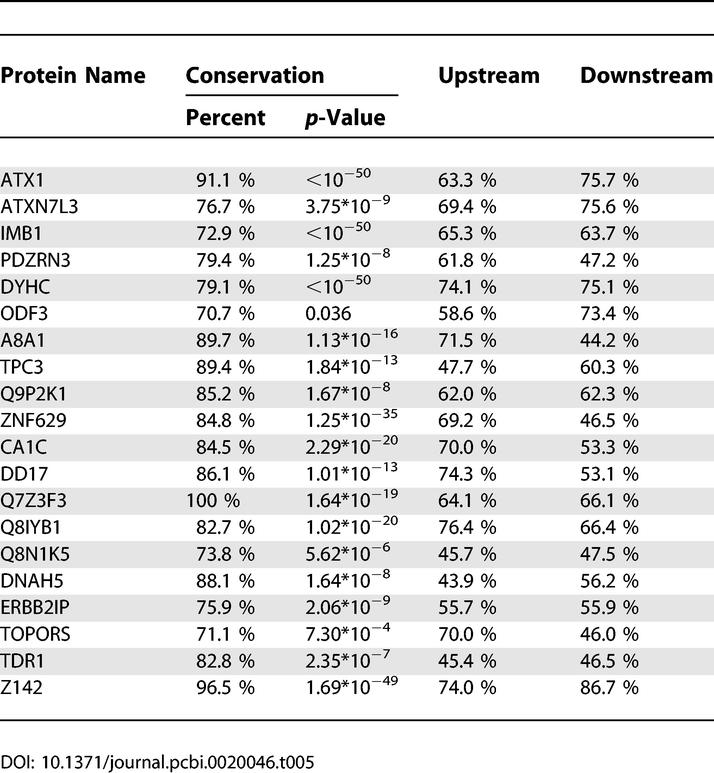
Conservation Percentage in and around the Pseudogene

Substitution rates vary along and between chromosomes. To make sure that it is the pseudogenes only, and not their genomic vicinities in general, that are conserved, we also aligned a 1,000-bp section upstream and downstream of each pseudogene. We observe in most cases (Table 5) that the conservation for the surroundings is about the expected 67% and much lower than for the actual pseudogene. The flanking sequences have in most cases about the 67% conservation that we expect. The unexpectedly high value registered downstream of, among others, the ATX1 relative, might be due to conservation of the 3′ UTR.

Conceivably, a potential pseudogene could in its close vicinity have protein-coding exons originating from the same gene. To exclude this possibility, we also checked the proximity for signs of exons originating from the same gene, with potentially intact protein-coding ability. No additional such protein-coding exons were found. For the absolute majority of our pseudogenes, no hit could be found on the same chromosome, and in no case was any hit found closer than 10,000 bp.

### Human–Chimpanzee Results

We also applied our methodology to the human–chimpanzee pair of genomes. This choice was motivated by our desire to discover young pseudogenes. Remember that the mouse Makorin pseudogene, although vital, has only been functional over a relatively short evolutionary period [7].

The procedure was the same as for human–mouse. The chimpanzee data was downloaded from Ensembl, including assembly 1 as of April 2005 together with protein sequences and gene-sequence data. For human–chimpanzee, sequence conservation is less effective as a means to separate functional from nonfunctional pseudogenes. The reason is of course that many pseudogenes originating before the comparatively recent primate species split can be expected to be nonfunctional, although they have not diverged sufficiently to be easily recognized as such. So, while in the human–mouse case we can be relatively confident that syntenic pseudogenes that prefer *S*1 are functional, it is likely that many *S*1 pseudogenes found in a human–chimpanzee comparison are nonfunctional. In fact, conservation estimates can, even together with expression evidence, be expected to be insufficient for revealing whether an individual pseudogene is functional or not. What we can hope for is a signal in the data showing that the quartets preferring *S*1 include functional pseudogenes.

As expected, the human–chimpanzee comparison resulted in a large set of pseudogene pairs. We therefore restricted our analysis to the most interesting class, i.e., class 1. We found 742 class 1 pseudogenes belonging to quartets favoring *S*1 (using *p*-value 0.001 for comparing *S*1 and *S*2 and 0.1 for comparing *S*1 and *S*3). The aforementioned class 1 pseudogenes found in the human–mouse comparison all belong to this set. We are fairly confident that these 742 sequences have indeed evolved in a manner atypical for a protein-coding gene. A key question is whether these are functional or not. Note that more than 1/5 of them show transcriptional evidence (Table 6).

**Table 6:**
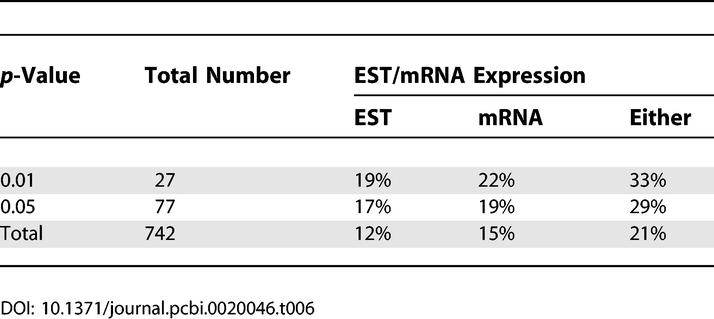
Percentage of Expressed Pseudogenes in Relation to Their Conservation *p*-Values (Calculated with Hoeffding's Bound)

Many pseudogenes have regulatory regions showing high similarity to those of the corresponding protein-coding genes. This is either because few mutations have occurred in these regions, or alternatively because many of the mutations that have occurred have been selected against, due to functionality of the pseudogenes.

To further purify our result set, i.e., the 742 pseudogenes favoring *S*1, we again looked at significant deviations of conservation. This approach requires a reliable estimate of the background conservation percentage and we used 5,000-bp-long alignments flanking each pseudogene to compute such an estimate.

Typical values for the background mismatch percentage range from 1.2% to 3% (counting the first but not subsequent indels in a gap), which conforms well with previous estimates of 1.4% [22] and 1.6% [23]. Note that the percentage of pseudogenes with EST and/or mRNA expression evidence is higher for more conserved pseudogenes. Table 7 contains a list of those pseudogenes that we have found to be expressed as well as conserved since the human–chimpanzee speciation. Notable is that the pseudogenes originating from proteins ATXN7L3, PDZRN3, or IMB1 are not found in Table 7. ATXN7L3 and PDZRN3 are not sufficiently conserved (in the former case we remember from the human–mouse analysis that it is only sections of the pseudogenes that exhibit exceptional conservation). The IMB1 pair residing on chromosome X is indeed conserved enough (it has a conservation *p*-value of 4.3*10^−4^) but lacks, as was noted in the human–mouse section, expression evidence.

**Table 7:**
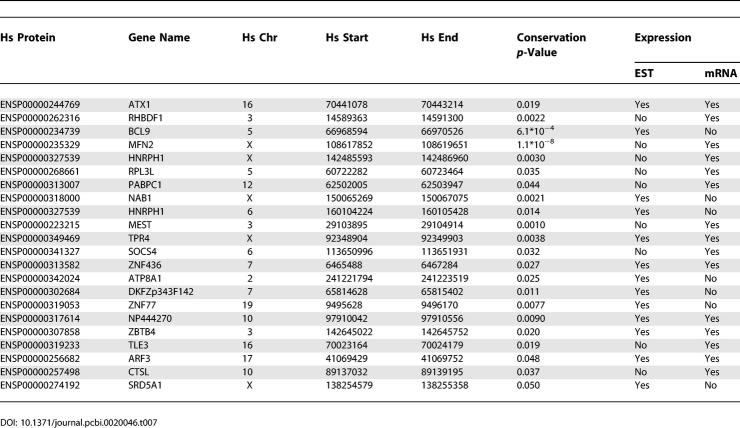
Human–Chimpanzee Conserved and Expressed Pseudogene Pairs

## Discussion

We have presented and applied a semi-automated methodology to identify pseudogenes of potential biological function. To the best of our knowledge, functional pseudogenes have never been observed in human. Our method uses no prior knowledge other than publicly available data on orthologous relationships for proteins, gene sequences, gene positions, and synteny maps.

The term *pseudogene* is normally used for sequences derived from known proteins but with detectable disablements that make the translation to protein impossible. Detecting pseudogenes is complicated by the possibility that part of the copy can be disabled, while the rest is coding.

We use conserved ancient pseudogenes as candidates of potential function. A computational approach based on support for four different evolutionary scenarios is used to obtain putative ancient pseudogenes. The *p*-value thresholds used, as well as the tests applied, indicate that the set of putative ancient pseudogenes is significantly enriched for ancient pseudogenes. It is interesting to ask whether there are evolutionary mechanisms that could cause our scenario *S*1 to appear more likely than another correct scenario, i.e., mechanisms not taken into account in our approach. Notice that, for instance, homogenization, e.g., through gene conversion, cannot make *S*1 more likely, since *S*1 is supported by similarity between sequences in *different* species.

We test functionality of our candidates by means of enrichment of synteny as well as of transcriptional activity and degree of conservation. We see, as expected, a clear overrepresentation of synteny for human–mouse pseudogene pairs originating before the species split. Interestingly, we also see tendencies for those examples that have evolved as pseudogenes since the species split to be both more abundantly expressed and more often syntenic than those that have not evolved as pseudogenes. For the latter finding, we believe that enrichment of functionality among our pseudogenes is the most likely explanation.

Judging from what is known from earlier work, the number of detectable pseudogenes originating from before the human–mouse speciation is limited. In [12], the authors found 11 examples of potentially orthologous pseudogene pairs. Although there is considerable overlap between their analysis and ours, a numerical comparison is not straightforward. First, they have stricter criteria for classifying a sequence as a pseudogene (deploying a careful filtering to make sure that only sequences that are processed pseudogenes are investigated). Second, they compute human and mouse orthologs using reciprocal BLAST comparisons only, and investigate how many of the transcribed human genes have mouse orthologs.

Three out of four (PDZRN3 being the exception) of our *S*1 examples belonging to class 1 are also classified as pseudogenes in [8,12]. We use the same databases for expression analyses as do the authors of [12], and, as expected, the results are in agreement.

To determine functionality of a human pseudogene, it is probably not sufficient to use information about whether it has a mouse ortholog or not, because many young pseudogenes can be found among orthologous pairs. Instead, we select only those human pseudogenes with orthologous mouse pseudogenes that satisfy the additional constraint that the least common ancestor was a pseudogene.

The results we present suggest that while functional pseudogenes are relatively rare on a long evolutionary timescale, they nevertheless exist. Our findings include a handful of sequences that are conserved since before the split of primates and rodents. Some of these are sequences predicted by gene finders to be protein-coding. We have found examples with, as well as without, detectable in-frame disablements. Apart from their apparent functional conservation and sometimes extensive expression activity, all these are poorly characterized. This can be due to the fact that some of the originating proteins are themselves not very well known or to the common assumption that pseudogenes are nonfunctional. Further characterization of these genes, their respective pseudogenes, and the interactions between them are areas for further studies.

We have noted with interest recent research activity concerning two of our top candidates, ATX1 and ATXN7L3. As is the case for the Ataxin gene family in general, these are associated with a number of neurodegenerative disorders primarily caused by expanded polyglutamine [24,25], but other than that their function is currently unknown [26]. Findings indicating that these genes have a regulating function [27,28] are of particular interest because it is reasonable to believe that their paralogs, if they are indeed functional pseudogenes, work on the RNA level. There are previously known examples of ncRNA genes in the Ataxin gene family. In [27] it is shown that Ataxin 8 regulates Kelch-like 1 by anti-sense regulation.

When extending the search to younger pseudogenes, i.e., applying our methodology to human–chimpanzee, the number of obtained pseudogenes is substantially larger than what was obtained in the human–mouse comparison. In this case, however, the assumption that nonfunctional pseudogenes originating before the speciation have diverged beyond recognition is not true. Consequently, filtering out nonfunctional pseudogenes is much harder than for the human–mouse case. Encouragingly, we found that the conservation of many pseudogenes is similar to that of nonsynonymous nucleotides in protein-coding genes (estimated to be 99.4% [23]).

There is an apparent tradeoff between the number of pseudogenes in the result set and the certainty with which we can state that they are functional. It is quite possible that both our choices of species pairs are in fact suboptimal, human–mouse being too evolutionarily distant and human–chimpanzee not distant enough. It will be interesting to apply our methodology on an intermediate timescale, and we plan to conduct a comparison between human and rhesus macaque.

## Materials and Methods

**Figure 10:**
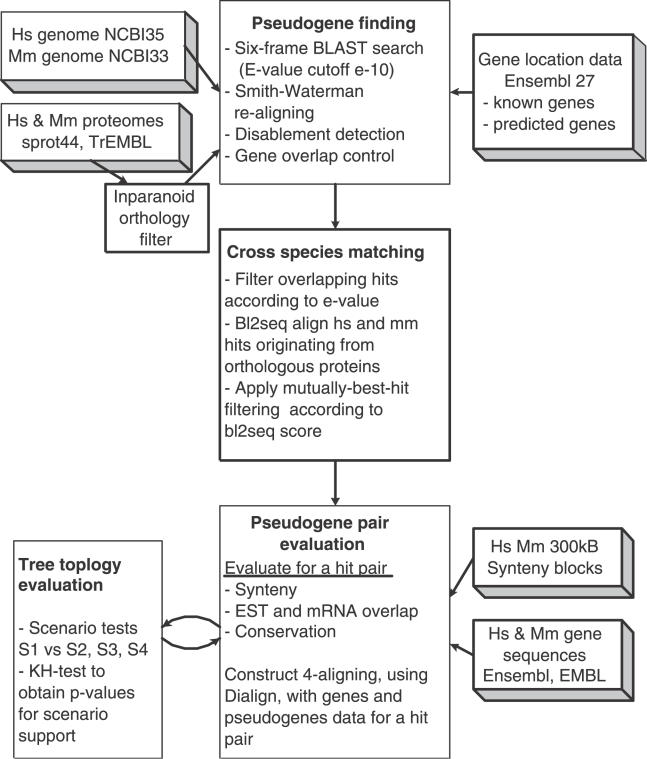
Flow Diagram over the Pseudogene Assignment Process

Our methodology includes three main parts (see Figure 10), here termed: 1) pseudogene finding, 2) cross-species matching, and 3) pseudogene pair evaluation.

To locate pseudogenes, we adopted a large part of the methodology presented in [8]. When searching for pseudogenes, a hit was considered to be significant if it had an e-value < 10^−10^ from a six-frame TBLASTN search with the protein sequence (BLAST-package was downloaded from ftp://ftp.ncbi.nih.gov/blast). We also made use of TBLASTN capabilities to detect stop codons and, importantly, sequence frameshifts.

We used repeat-masked genomic sequence data NCBI35 (human) and NCBIm33 (mouse) downloaded in January 2005 from the Ensembl database version 27. The Expasy protein database (sprot44, human_trembl and rodent_trembl) was used to assemble protein sequence sets for the two species. We used the Inparanoid [17] database to retrieve orthologous protein pairs, by selecting from each set of inparalogs the sequence with highest score.

The mutual-best-hit filtering was performed for each pair of orthologous proteins, by aligning each pair of pseudogenes from the respective species, using bl2seq (from the BLAST package) and then selecting the pair with the best score. We aligned the thereby obtained quartets using the Dialign package [29], and we extracted from the Dialign output gap-free column triplets based on the reading frame induced by the genes. By using a *local* alignment program we reduced the risk of misalignments caused by introns in the pseudogenes. 

To select the scenario (*S*1, *S*2, *S*3, *S*4) that best describes a given quartet, we adapted the method outlined in [30]. We work with a model describing the instantaneous substitution rate from codon *i* to codon *j, q_ij_*, given that the equilibrium frequency of codon *j* is *π_j_*. In their model, the substitution rates are specified by the instantaneous rate matrix *Q* = {*q_ij_*} defined by:

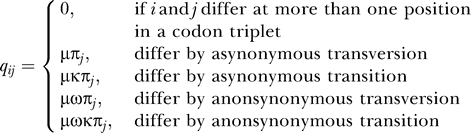
where *μ* is a normalizing rate factor, *κ* is the transition/transversion ratio, and ω is the nonsynonymous-to-synonymous ratio. In our model, we use one matrix, *Q_g_,* for the parts of the tree where the sequences are supposed to evolve as a gene, and another matrix, *Q_ψ_,* for the parts of the tree where the sequences are supposed to evolve as a pseudogene. The difference between the matrices is that different *κ* are used, for pseudogenes *ω* equals one, for genes we do not allow transitions to stop codons, and equilibrium frequencies are estimated from gene or pseudogene sequences, respectively.

We used the nonparametric version of the Kishino–Hasegawa bootstrap test with 1,000 bootstraps to obtain *p*-values for scenario support [31].

### Synteny evaluation.

To infer whether two pseudogenes are in synteny, we used synteny maps from [32]. Maps based on synteny blocks of a minimum size of 300 kB were downloaded from http://www.cse.ucsd.edu/groups/bioinformatics/GRIMM. Pairs of pseudogenes were annotated according to: 1) syntenic, i.e., the two sequences originate from syntenic regions; 2) reversed syntenic, the same as above, but their mutual orientation differs from the main orientation of the synteny blocks; 3) close to synteny, the sequence from one species is found in a block adjacent to the syntenic block in the other species; 4) undetermined synteny, one or both sequences originate from positions which are not mapped to any synteny block; and 5) not in synteny.

Synteny relations were established for the 7,244 out of 12,678 genes for which gene position data was available.

### Gene expression evaluation.

To find transcription evidence we applied a reciprocal BLAST-based methodology to databases of ESTs and mRNAs. The EST-human, EST-mouse, and Unigene mRNA databases were downloaded from NCBI. Any reciprocal best hit longer than 100 bp and with more than 99% sequence identity to the query sequence was retrieved.

### Hoeffding's bound for calculation of conservation *p*-value.

According to Hoeffding's theorem [33], the following holds: given a set of *n* Poisson trials *X_i_,* each taking value one with probability *p_i_,* and *X =* ∑ *X_i_,* with expectation *E*[*X*] = *np,* it holds that *Pr*(*X ≤ c*) ≤ *Bin*(*n,p,c*) for any 0 ≤ c ≤ (*np* − 1).

Given an alignment of length *n,* the theorem can be used to calculate a *p*-value for ≥ *c/n* matching residues based on the hypothesis that the alignment is generated from a (background) distribution with mismatch probability *p*.

## Abbreviations

ATX1: Spinocerebellar ataxia type 1 protein
ATX7NL3: Ataxin 7-like 3
EST: expressed-sequence tags

## References

[pcbi-0020046-b001] Vanin EF (1985). Processed pseudogenes: Characteristics and evolution. Annu Rev Genet.

[pcbi-0020046-b002] Mighell AJ, Smith NR, Robinson PA, Markham AF (2000). Vertebrate pseudogenes. FEBS Lett.

[pcbi-0020046-b003] Graur D, Shuali Y, Li (1989). Deletions in processed pseudogenes accummulate faster in rodents than in humans. J Mol Evol.

[pcbi-0020046-b004] Zhang Z, Gerstein M (2003). Patterns of nucleotide substitution, insertion and deletion in the human genome inferred from pseudogenes. Nucleic Acids Res.

[pcbi-0020046-b005] Hirotsune S Yoshida N Chen A Garrett L Sugiyama F> 2003 An expressed pseudogene regulates the messenger-RNA stability of its homologous coding gene Nature 423 91 96 12721631 10.1038/nature01535

[pcbi-0020046-b006] Balakirev ES, Ayala FJ (2003). Pseudogenes: Are they “junk” or functional DNA?. Annu Rev Genet.

[pcbi-0020046-b007] Podlaha O, Zhang J (2004). Nonneutral evolution of the transcribed pseudogene Makorin1-p1 in mice. Mol Biol Evol.

[pcbi-0020046-b008] Zhang Z, Harrison PM, Liu Y, Gerstein M (2003). Millions of years of evolution preserved: A comprehensive catalog of the processed pseudogenes in the human genome. Genome Res.

[pcbi-0020046-b009] Torrents D, Suyama M, Zdobnov E, Bork P (2003). A genome-wide survey of human pseudogenes. Genome Res.

[pcbi-0020046-b010] Zhang Z, Carriero N, Gerstein M (2004). Comparative analysis of processed pseudogenes in the mouse and human genomes. Trends Genet.

[pcbi-0020046-b011] Ohshima K, Masahira H, Yada T, Gojobori T, Sakaki Y (2003). Whole-genome screening indicates a possible burst of formation of processed pseudogenes and Alu repeats by particular L1 subfamilies in ancestral primates. Genome Biol.

[pcbi-0020046-b012] Harrison PM, Zheng D, Zhang Z, Carriero N, Gerstein M (2005). Transcribed processed pseudogenes in the human genome: An intermediate form of expressed retrosequence lacking protein-coding ability. Nucleic Acids Res.

[pcbi-0020046-b013] Zheng D, Zhang Z, Harrison P, Karro J, Carriero N, et al. (2005). Integrated pseudogene annotation for human chromosome 22: Evidence for transcription. J Mol Biol.

[pcbi-0020046-b014] Yano Y, Saito R, Yoshida N, Yoshiki A, Wynshaw-Boris A, et al. (2004). A new role for expressed pseudogenes as ncRNA: Regulation of mRNA stability of its homologous coding gene. J Mol Med.

[pcbi-0020046-b015] Fleishman SJ, Dagan T, Graur D (2003). pANT: A method for the pairwise assessment of nonfunctionalization times of processed pseudogenes. Mol Biol Evol.

[pcbi-0020046-b016] Elhaik E, Sabath N, Graur D (2006). The “inverse relationship between evolutionary rate and age of mammalian genes” is an artifact of increased genetic distance with rate of evolution and time of divergence. Mol Biol Evol.

[pcbi-0020046-b017] Remm M, Storm CE, Sonnhammer EL (2001). Automatic clustering of orthologs and in-paralogs from pairwise species comparisons. J Mol Biol.

[pcbi-0020046-b018] Pevzner P, Tesler G (2003). Genome rearrangements in mammalian evolution: Lessons from human and mouse genomes. Genome Res.

[pcbi-0020046-b019] Korf I, Flicek P, Duan D, Brent MR (2001). Integrating genomic homology into gene structure prediction. Bioinformatics.

[pcbi-0020046-b020] Malcom CM, Wyckoff GJ, Lawn BT (2003). Genic mutation rates in mammals: Local similarity, chromosomal heterogeneity, and X-versus-autosome disparity. Mol Biol Evol.

[pcbi-0020046-b021] Waterston RH, Lindblad-Toh K, Birney E, Rogers J, Abril JF, et al. (2002). Initial sequencing and comparative analysis of the mouse genome. Nature.

[pcbi-0020046-b022] Britten RJ (2002). Divergence between samples of chimpanzee and human DNA sequences is 5%, counting indels. Proc Natl Acad Sci U S A.

[pcbi-0020046-b023] Wildman DE, Uddin M, Guozhen L, Grossman L, Goodman M (2003). Implications of natural selection in shaping 99.4% nonsynonymous DNA identity between humans and chimpanzees: Enlarging genus Homo. Proc Natl Acad Sci U S A.

[pcbi-0020046-b024] Orr HT, Chung MY, Banfi S, Kwiatkowski TJ Jr, Servadio A, et al. (1993). Expansion of an unstable trinucleotide CAG repeat in spinocerebellar ataxia type 1. Nat Genet.

[pcbi-0020046-b025] Banfi S, Servadio A, Chung MY, Kwiatkowski TJ, McCall AE Identification and characterization of the gene causing type 1 spinocerebellar ataxia. Nat Genet.

[pcbi-0020046-b026] Tsai CC, Kao HY, Mitzutani A, Banayo E, Rajan H Ataxin 1, a SCA1 neurodegenerative disorder protein, is functionally linked to the silencing mediator of retinoid and thyroid hormone receptors. Proc Natl Acad Sci U S A.

[pcbi-0020046-b027] Nemes JP, Benzow KA, Koob MD (2000). The SCA8 transcript is an antisense RNA to a brain-specific transcript encoding a novel actin-binding protein (KLHL1). Hum Mol Genet.

[pcbi-0020046-b028] Ström AL, Forsgren L, Holmberg M (2005). A role for both wild-type and expanded ataxin-7 in transcriptional regulation. Neurobiol Dis.

[pcbi-0020046-b029] Morgenstern B (1999). DIALIGN 2: Improvement of the segment-to-segment approach to multiple sequence alignment. Bioinformatics.

[pcbi-0020046-b030] Bielawski JP, Yang Z (2003). Maximum likelihood methods for detecting adaptive evolution after gene duplication. J Struct Funct Genomics.

[pcbi-0020046-b031] Goldman N, Anderson JP, Rodrigo AG (2000). Likelihood-based tests of topologies in phylogenetics. Syst Biol.

[pcbi-0020046-b032] Bourque G, Pevzner P, Tesler G (2004). Reconstructing the genomic architecture of ancestral mammals: Lessons from human, mouse, and rat genomes. Genome Res.

[pcbi-0020046-b033] Hoeffding W (1956). On the distribution of the number of successes in independent trials. Ann Math Stat.

[pcbi-0020046-b034] Beitz E (2000). TeXshade: Shading and labeling of multiple sequence alignments using LaTeX2e. Bioinformatics.

